# Uptake of Preventive Treatment for Intestinal Schistosomiasis among School Children in Jinja District, Uganda: A Cross Sectional Study

**DOI:** 10.1371/journal.pone.0063438

**Published:** 2013-05-07

**Authors:** Simon Muhumuza, Annette Olsen, Anne Katahoire, Fred Nuwaha

**Affiliations:** 1 Makerere University, School of Medicine, Child Health and Development Center, Kampala, Uganda; 2 University of Copenhagen, Faculty of Health and Medical Sciences, Section for Parasitology, Health and Development, Copenhagen, Denmark; 3 Makerere University, School of Public Health, Kampala Uganda; Université Catholique de Louvain, Belgium

## Abstract

**Background:**

In Uganda, the current national health sector strategic and investment plan underscores schistosomiasis as one of the diseases targeted for elimination by the year 2015. However, uptake of treatment among school children is unknown but suspected to be low. We estimated the uptake and predictors of preventive treatment with praziquantel.

**Methods:**

In a cross sectional study carried out in Jinja district of Uganda, a random sample of 1,010 children in 12 primary schools was questioned about their uptake of praziquantel, knowledge and perceptions about schistosomiasis, support for taking preventive treatment and the dangers of taking praziquantel. The prevalence and mean intensity of infection with *Schistosoma mansoni* were determined.

**Results:**

Self reported uptake of praziquantel at last mass treatment was 28.2% (95% confidence interval (CI): 22.9%–33.6%). Overall prevalence and mean intensity of *S. mansoni* infection was 35% (95% CI: 25.4%–37.9%) and 116.1 eggs per gram (epg) of stool (95% CI: 98.3–137.1) respectively. Uptake of praziquantel was more likely if a child was from a school with high prevalence of infection, had knowledge about schistosomiasis transmission and prevention, and reported teachers’ support to take praziquantel. Of the 285 children who took praziquantel, 142 (49.8%) developed side effects. Of the 725 children who did not take the drug, 522 (72.0%) reported fear of side effects as a major reason for non-uptake.

**Conclusions:**

Uptake of praziquantel in this population is very low. Fear of side effects of praziquantel, lack of knowledge about schistosomiasis transmission and prevention and lack of teacher support are some of the major factors associated with low uptake.

## Introduction

Globally, an estimated 207 million people are infected with schistosomiasis and more than 700 million people are at risk of infection in 76 countries [1], [2], [3]. 90% of the global schistosomiasis burden is shouldered by low income countries of sub-Saharan Africa [1], [3], [4] with Uganda described as the cradle of *Schistosoma mansoni* in the region [5]. The infection is particularly more important in school-age children, where, if left untreated, results in retarded growth and impaired cognitive function [2], [6]. Therefore, repeated treatment in the early stages of life has a long-lasting effect on morbidity at a later age [7], [8]. The major control strategy for schistosomiasis is preventive treatment with praziquantel [9], [10], [11], [12]. Since 2001, when WHO recommended that regular praziquantel treatment against schistosomiasis should be provided to 75% of school-age children in high burden regions by 2010, there has been increased efforts and financing for schistosomiasis control [13]. Recently, an additional fivefold increase in support for programs to control neglected tropical diseases (NTDs) were announced by the United Kingdoms’ department for International Development to support delivery of 100 million treatments for schistosomiasis [14]. The main activities supported under these initiatives include mapping of the most heavily infected regions in each of the target countries, training of local health staff to effectively manage the infection, sensitization of affected communities on prevention activities, and treatment of infected individuals with efficacious medicines; praziquantel for schistosomiasis and albendazole or mebendazole for soil-transmitted helminths (STH) [10], [12], [15]. In Uganda, the national program for the control of schistosomiasis and other NTDs adopted the WHO recommendation in March 2003 and has since supported mass drug administration (MDA) with praziquantel and albendazole. By the fourth year of the control program, over 28 million treatments had been provided to more than 13 million people in 72 districts within the country (http://ntd.rti.org, accessed on 29^th^ November 2012). The current national health sector strategic and investment plan underscores schistosomiasis as one of the diseases targeted for elimination by the year 2015 [16]. The plan further indicates that integrated MDA against onchocerciasis, schistosomiasis, lymphatic filariasis and STH is ongoing and has been scaled to most endemic districts [17], [18], [19].

In Jinja district, implementation of the schistosomiasis control program started in August 2003. The district vector control officer under the district health office is the focal person for the program. The district cascades training and supervision duties to the sub-county supervisors who comprise of health workers and health assistants and training of the community drug distributors and teachers. The main activities of the control program include preventive measures focusing on raising awareness on schistosomiasis through distribution of Information, Education and Communication materials and health education especially in the primary schools but also in the wider communities. These are provided prior to MDA. Mass treatment with praziquantel and albendazole is given to all primary school children and adults at risk of the infection. These activities are supported through a parallel structure within the Ministry of Health with external funding from the United States Agency for International Development (USAID) channeled through Research Triangle Institute (RTI) International.

Previous research undertaken among adults in high endemic districts of Busia, Adjumani, Moyo and Nebbi reported unwillingness to take preventive treatment [18], [19], [20]. Uptake of praziquantel among school children is not known. The objective of the current study is to report on levels of and predictors of uptake of preventive treatment for intestinal schistosomiasis among primary school children.

## Materials and Methods

### Study Design

This was a cross-sectional survey conducted in September 2011, six months after MDA. The survey employed quantitative methods of data collection.

### Study Setting

The study was conducted in Walukuba Division, Jinja District, South Eastern Uganda. *Schistosoma mansoni* is highly endemic in the Division with a prevalence of 65% among school age children [17]. Lake Victoria which borders the Division to the south is the main source of *S. mansoni* infection. The Division has a total population of 40,882. The main socio-economic activities in the area include agriculture (subsistence farming), fishing, and petty trade between the main land and the islands. There are 12 primary schools in the Division, majority (8/12) of which are within a 5 km distance from the Lake.

#### Implementation of MDA in the primary schools

MDA in the primary schools in the Division is implemented on an annual basis as a standalone intervention. School teachers in-charge of health and sanitation are the focal persons for MDA. Prior to MDA with praziquantel and albendazole, the grade teachers sensitize their respective children about schistosomiasis prevention, including taking preventive treatment, mobilize and prepare them to receive treatment. This is done on a group (grade) basis. During MDA, a classroom in each school is organized for drug administration and all children are invited indiscriminately, according to their grade, to receive treatment. One grade is invited at a time. Praziquantel is distributed according to height of the child using a standard dose pole. In addition, each child receives a single tablet of albendazole. Both drugs are distributed by the teachers and the children swallow the tablets using water under observation of the teachers who also record the treatment in the registers. After MDA, the health assistant in-charge of the division collects the remaining drugs and the filled registers and delivers them to Walukuba health center IV where they are kept for accountability and reporting purposes. During the 2011 MDA, only praziquantel was distributed because albendazole was out of stock.

### Sample Size

The sample size used in this study was based on that required to determine the difference in uptake of treatment among children in a community setting and that in primary schools. The uptake used in this study was 42% from a previous study in a community setting [17]. It was assumed that uptake of treatment in primary schools is lower by 5%. At a 90% power and a 95% confidence interval, the sample size needed to detect this difference according to STATA 10.0 (TX, USA) is 1,007. The STATA command used is “sampsi 0.42 0.37, power (0.9) onesample”.

### Sampling and Data Collection

Children in grade (year) 4–6 in the 12 primary schools were randomly selected to participate in the study. This is because children in grade 4–6 are about 10–14 years of age which is the peak age for schistosomiasis infection in Uganda [5]. A proportionate number of children selected from each school and grade was determined by probability proportional to size of the school and grade population. Systematic sampling, using the grade registers as the sampling frame, where the names of the children are arranged in alphabetical order, was employed. The sampling interval was obtained by dividing the total population of the grade with the number of children to be studied in the grade (N/n). After obtaining a random start from a table of random numbers, the interval was followed until the required number of children in each grade was obtained. Face to face interviews with each selected child were conducted by trained research assistants using structured questionnaires with multiple choice questions. After the interview, stool specimens were collected from each child, processed and examined for *S. mansoni* infection. Children who failed to provide stool specimens for examination were replaced by randomly selecting an equal number of children in grade 4–6. A total of 20 children did not provide stool specimens and were replaced. The data was made available and shared with the national Vector Control Division, Ministry of Health.

### Study Variables

In this study, uptake of preventive treatment was defined as having received and swallowed the drug at the last MDA. This was measured through self report. The independent variables included i) demographic variables such as school, grade (year), age, sex, distance of residence/school from the lake, ii) Individual factors such as knowledge of schistosomiasis transmission and prevention, perceived risk to infection, perceptions about schistosomiasis, water contact patterns and self-efficacy towards treatment with praziquantel, iii) social/economical factors such as water sources in homes/schools, sanitation facilities, iv) side effects of praziquantel and iv) interpersonal relationships including family support, teacher support, peer support and health worker support. Knowledge of schistosomiasis focused on the children’s understanding of how the disease is transmitted and how it can be prevented. Different ways through which the disease can be acquired and prevented were listed on the questionnaire and a child who mentioned at least one correct method of transmission and one correct method of prevention was regarded as having correct knowledge of schistosomiasis transmission and prevention. Children were further asked whether they think schistosomiasis is a problem in their area and whether they are at risk of acquiring the infection. Children were also asked if it is important to take preventive treatment for schistosomiasis. They were further asked whether they think that their families, teachers, peers and health workers support preventive treatment. Those who received praziquantel at the last MDA were asked if they developed any side effects after swallowing the drug. The reasons for non-uptake among those who did not take the drugs were also established through multiple choice questions with an option of “others (please specify)” in case a child mentioned a reason that is not captured on the questionnaire.

#### Laboratory diagnosis of S. mansoni infection

We examined one stool specimen from each child on two consecutive days using the Kato-Katz faecal thick smear technique as modified by Katz *et al.* (1972) [21]. Two (2) slides per stool specimen were prepared and examined by two independent laboratory technicians. A discrepancy of more than 5% in egg count was validated by a third laboratory technician who read the results for confirmation. Eggs of *S. mansoni* were counted and their arithmetic mean recorded on a result sheet. This was multiplied by 24 to obtain the number of eggs per gram (epg) of stool. Because egg counts are over dispersed, they were logarithmically transformed into a geometric mean (GM). In case of the presence of STH infections on the slides, this was registered so the child could be treated.

### Statistical Analysis

Analysis was done at the school level to obtain school-specific uptake of praziquantel, prevalence and intensity of *S. mansoni* infection. Mean and standard deviation (S.D) were used to describe continuous data. Bivariate analysis of the various variables was done to identify factors associated with uptake of praziquantel using crude odds ratios (COR) and their 95% confidence interval (CI). Multivariable logistic regression was done to identify the independent predictors of uptake of praziquantel. Within-school clustering was adjusted for using the cluster robust option and robust standard errors were used. Missing data was checked for by comparing variables between the subjects with missing data and those with complete data and there were no significant differences. It was assumed the data was missing completely at random. STATA 10.0 (TX, USA) was used for analysis.

### Quality Control

All the study primary schools were pre-visited to obtain updated sampling frames. We recruited experienced research assistants who could speak the local language. These were trained in data collection methods. The questionnaires were pre-tested in one of the schools in a non-study area for purposes of clarity, validation, suitability and logical flow of the questions. Questionnaire data was checked for completeness and accuracy before leaving the school premises. The research assistants were closely supervised by the principal investigator during data collection.

### Ethical Considerations

We obtained ethical permit for the study from Makerere University College of Health Sciences Higher Degrees, Research and Ethics Committee and the Uganda National Council for Science and Technology. Permission to conduct the study in the schools was obtained from the school management. Further, we obtained consent from parents of the children selected to participate in the study followed by assent from the children. Children identified with schistosomiasis and STH were treated with praziquantel and albendazole. Confidentiality was maintained by use of a coding system.

## Results

A total of 1,010 children in grade 4, [484 (47.9%)], grade 5 [268 (26.5%)] and grade 6 [258 (25.5%)] in 12 primary schools were enrolled. The mean age was 11.6 years (S.D 1.8). Females accounted for 55.0% (555). The majority (>73%) lived in households and attended schools that are within a 5 km radius from Lake Victoria. The overall uptake of praziquantel was 28.2% [95% CI: 25.4%–30.9%]. The intracluster correlation coefficient (ICC) was 0.06. There was a considerable variation in uptake of praziquantel across the primary schools that ranged from 11.2% to 69.0%. The overall prevalence of *S. mansoni* infection was 35.0% [95% CI: 32.1%–37.9%]. The prevalence also varied across the schools and ranged from 16.3% to 96.8%. The majority 6 (50%) of the schools had moderate prevalence while 3 (25%) had high prevalence and the other 3 (25%) had low prevalence, according to WHO classification [22]. The overall intensity of *S. mansoni* infection was 116.1 epg [95% CI: 98.3–137.1]. The intensity of the infection ranged from 43.5 to 668.6 epg of stool (Table 1).

**Table 1:** Self-reported uptake of praziquantel, prevalence and intensity of *S. mansoni* infection in the 12 primary schools.

School (No)	Childrenexamined	Uptake%, (95% CI)	Prevalence%, (95% CI)	Intensity*GM, (95% CI)
1.	70	17.1 (2.08–48.4)	24.3 (14.8–36.0)	93.1 (52.0–169.5)
2.	61	26.2 (7.27–52.3)	19.7 (10.5–31.8)	81.7 (46.0–145.0)
3.	75	25.3 (9.14–51.2)	36.0 (25.2–47.9)	57.7 (37.4–89.1)
4.	55	69.1 (51.3–82.4)	94.5 (84.8–98.9)	668.6 (472.8–945.5)
5.	31	64.5 (40.7–84.6)	96.8 (83.2–99.9)	639.7 (367.8–1112.6)
6.	104	25.0 (11.5–47.8)	55.8 (45.6–65.5)	84.1 (58.3–121.6)
7.	107	23.4 (9.35–45.1)	35.5 (26.5–45.3)	69.6 (43.7–110.8)
8.	107	11.2 (0.21–38.4)	30.0 (21.4–39.5)	89.5 (59.0–135.9)
9.	82	58.5 (43.2–72.4)	34.1 (24.0–45.4)	46.3 (26.5–80.8)
10.	104	16.3 (3.70–43.4)	19.2 (12.1–28.1)	85.6 (42.3–173.4)
11.	110	25.5 (10.6–44.9)	21.0 (13.7–29.7)	43.5 (31.9–59.3)
12.	104	23.1 (9.77–46.7)	16.3 (9.80–24.8)	46.7 (31.8–68.3)
**Total**	**1010**	**28.2 (22.9–33.6)**	**35.0 (25.4–37.9)**	**116.1 (98.3–137.1)**

* GM (Geometric mean) is among the positive cases only.

### Predictors of Uptake of Praziquantel

#### Side effects of praziquantel

The side effects explored included abdominal pain, diarrhoea, vomiting, headache and dizziness. These are the most common reported side effects of praziquantel [18], [19], [23]. Of the 285 children who swallowed the drug, 142 (49.8%) reported to have developed at least one side effect. The majority 124 (87.3%) reported abdominal pain, 34 (23.9%) developed diarrhoea, 21 (14.8%) vomited and 3 (2.1%) reported to have developed a headache. Of the 725 children who did not take the drug, 522 (72.0%) reported fear of side effects of praziquantel as a major reason for non-uptake. Of these, 337 (72.3%) were deliberately absent from school during MDA due to fear of side effects. Furthermore, 753 (74.6%) of all interviewed children reported that they would not swallow the drug during the subsequent MDA due to fear of side effects.

#### Prevalence of *S. mansoni* infection

**Figure 1:**
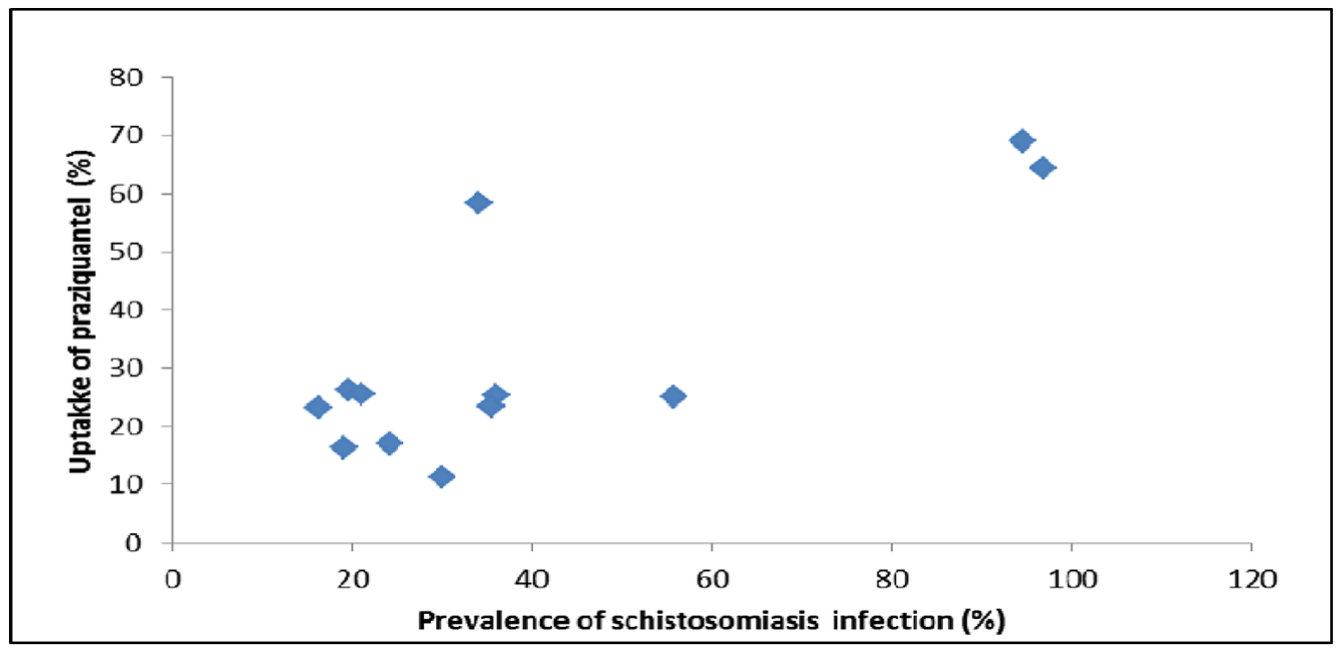
Comparison between uptake of praziquantel and prevalence of *S.* *mansoni*.

Uptake of praziquantel in schools with higher prevalence and intensity of infections was generally much higher than in the schools with low prevalence and intensity infection as is shown in Figure 1.

#### Independent predictors of uptake of praziquantel

Through a step wise backward elimination method, variables that were associated with uptake of praziquantel at bivariate level (p<0.05) (knowledge of schistosomiasis transmission and prevention, lake water as main source of water for domestic use, likelihood to take praziquantel at the subsequent MDA, parental and teacher support to take preventive treatment) were considered for inclusion in the multivariable model. The variables retained in the final model were knowledge of schistosomiasis transmission and prevention and teacher support to take preventive treatment (Table 2).

**Table 2:** Crude odds ratios (COR) and Adjusted odds ratio (AOR) and their 95% confidence interval (CI) for predictors of uptake of praziquantel.

Variable	Uptake of Praziquantel (%)	COR (95% CI)	P-value	AOR (95% CI)	P-value
Lake water as main source of water for domestic use					
Yes	204 (25.5)	1.23 (1.13–1.43)	<0.001	–	–
No	596 (74.5)	1.00			
Likely to take praziquantel at subsequent MDA					
Yes	119 (24.3)	1.31 (1.05–1.65)	0.02	–	–
No	370 (75.7)	1.00			
Parental support to take preventive treatment					
Yes	279 (29.7)	5.56 (2.27–14.3)	<0.001	–	**–**
No	659 (70.3)	1.00			
Teacher support to take preventive treatment					
Yes	279 (30.0)	5.26 (2.44–11.1)	<0.001	2.63 (1.25–5.55)	**0.01**
No	650 (70.0)	1.00			
Knowledge of schistosomiasis transmission and prevention					
Yes	175 (37.8)	2.44 (1.45–4.00)	0.001	2.04 (1.23–3.45)	**0.01**
No	288 (62.2)	1.00			

## Discussion

This study found that less than one third of the primary school children took preventive treatment for schistosomiasis at the last MDA. Fear of side effects of praziquantel, lack of knowledge of schistosomiasis transmission and prevention and lack of teacher support to take preventive treatment are some of the factors associated with the low uptake of praziquantel among primary school children.

The self-reported uptake of praziquantel among primary school children in this study is way far below the WHO target [13], [24]. Further still, this uptake is lower than that reported among adults in Moyo district (41%) in 2007 and in Busia district (50%) in 2009 [18]. A comparable low coverage among primary school children (28.5%) was reported in rural communities in Nigeria in an evaluation of different approaches to mass delivery of praziquantel among school-aged children [25]. The low uptake of preventive and treatment services do not only apply to schistosomiasis control but also to the control of malaria [26], tuberculosis [27], [28] and other tropical diseases [29].

Because schools are considered to be widely distributed, it is argued that it is cost-effective to deliver interventions through schools [4], [30], [31] and feasible to provide regular treatment with praziquantel to at least 75% of school-age children at risk of morbidity [24]. However, fear of side effects of praziquantel, reliance on volunteers to distribute drugs, ineffective communication about the rationale for MDA and poor monitoring and evaluation of MDA programs have been highlighted as major challenges facing the control program [4], [18], [29]. The findings in this study also suggest that the current MDA approach may not achieve its objective. The widespread concern about the side effects of praziquantel cannot be ignored. Side effects attributable to praziquantel were reported in 80% of the treated population in an earlier study in Uganda. The report however, assumes that these did not pose any serious or long-term complications to affect compliance to treatment [9]. A related study among pre-school children reported comparable side effects with an amelioration within 21 days of follow up [23]. Praziquantel is commonly perceived as an aggressive drug that causes transient sickness and occasional fatalities [18], [19], [20], [32]; the severity of side effects of praziquantel unquestionably generates a lot of apprehension and aversion towards swallowing the drug [19]. In this study, half of the children who swallowed praziquantel reported to have developed at least one major side effect. More than 72% of the children who did not take the drug reported fear of side effects as a major reason for non uptake, the majority of whom were deliberately absent from school during MDA. Therefore, focusing on the narrow logistical aspects of drug delivery is not enough. Implementing measures for mitigating side effects attributable to praziquantel, such as providing a snack prior to MDA may improve uptake of the drug among school children.

In this study, less than a half of the children had knowledge of schistosomiasis transmission and prevention. There is no doubt that health education facilitates a better understanding of the obvious risks to health, including the knowledge of preventing parasitic infections among primary school children. The reverse is true in situations where such knowledge is seen to be lacking [33]. Better compliance to treatment for schistosomiasis among school children can be achieved through implementing carefully designed programs involving face to face education methods [34], [35], [36]. Otherwise, it is perilous to presume that the targeted populations will accept treatment if they are not adequately educated about the infection and rationale for preventive treatment.

Children’s behaviour can be potentially shaped by the closest social circles including peers, partners and family members with which the individual interacts [37]. In primary schools, teachers play a key role in promoting behavioral change among children. In relation to MDA, teachers sensitize and mobilize the children to receive treatment. In addition, they distribute and record the treatment. Children in the study area frequently visit the lake to fetch water, bathe, to wash, to fish and to swim [17] and therefore get infected with schistosomiasis when they come into contact with contaminated water. Aware of the increased likelihood of acquiring the infection, teachers from schools which are close to the lake and have high prevalence of *S. mansoni* infection are more supportive and tend to encourage their children to take preventive treatment compared to their counterparts from schools that are distant from the lake where the infection is not perceived as a public health problem. In this study, 25% of schools had low prevalence infections. Screening of all school children and treatment of the positive cases is the recommended measure in such schools with low prevalence [22]. One would therefore question the rationale of indiscriminate administration of praziquantel to individuals who are not infected in these low prevalence schools, more especially when the drugs cause undesirable effects.

### Study Limitations

Three limitations in this study are worth mentioning. First, data on self-reported uptake were derived from interviews. It is possible that the children were unable to recall treatment. Nonetheless, a number of relationships corroborate evidence that self-report in this study is fairly accurate. Majority of the children clearly recalled treatment because praziquantel tablets are unusually large, pungent and unpalatable [32]. For instance, among those who reported uptake of praziquantel, 257 (90.2%) were able to recall having swallowed the drug, 257 (90.2%) were able to recall and mention the true colour of praziquantel and 262 (91.9%) were able to recall whether they experienced side effects with the drug or not. Secondly, the high prevalence and intensity of *S. mansoni* infections reported may be due to re-infection since the study was conducted six months after MDA. Nonetheless, one does not expect the prevalence and intensity to go down with low uptake of praziquantel. The third limitation is the relatively low intrinsic capacity of the Kato-Katz technique to recover parasite eggs from specimens of low infection intensities compared to other parasitological methods [38], [39]. To increase its sensitivity during the study, two (2) slides per stool specimen were prepared.

### Conclusion

This study reveals that the current MDA approach may not achieve the stated objective of the national health sector strategic and investment plan of eliminating the infection by 2015. There is need to establish more effective drug delivery strategies in the primary schools. Periodic screening of school children and targeted treatment will be an essential step in minimizing drug wastages and a possible emergence of resistance to treatment if praziquantel is continually distributed to individuals who do not need it. Increasing teacher support and implementing measures to mitigate the side effects attributable to praziquantel, such as providing a snack prior to drug administration may improve uptake of the drug among school children. Undertaking a more in-depth anthropological study to further explore the underlying perceptions and beliefs towards praziquantel among school children is needed.
